# Innate immune responses in pneumonia

**DOI:** 10.1186/s41479-023-00106-8

**Published:** 2023-02-25

**Authors:** Filiz T. Korkmaz, Katrina E. Traber

**Affiliations:** 1 Department of Medicine, Division of Immunology & Infectious Disease, University of Massachusetts, Worcester, MA USA; 2 Pulmonary Center, Boston University School of Medicine, Boston, MA USA; 3 Department of Medicine, Boston University School of Medicine, Boston, MA USA

**Keywords:** Innate immunity, Viral pneumonia, Bacterial pneumonia, Macrophage, Neutrophil, Innate lymphocytes, Lung epithelium, Lung endothelium, Extracellular matrix

## Abstract

The lungs are an immunologically unique environment; they are exposed to innumerable pathogens and particulate matter daily. Appropriate clearance of pathogens and response to pollutants is required to prevent overwhelming infection, while preventing tissue damage and maintaining efficient gas exchange. Broadly, the innate immune system is the collection of immediate, intrinsic immune responses to pathogen or tissue injury. In this review, we will examine the innate immune responses of the lung, with a particular focus on their role in pneumonia. We will discuss the anatomic barriers and antimicrobial proteins of the lung, pathogen and injury recognition, and the role of leukocytes (macrophages, neutrophils, and innate lymphocytes) and lung stromal cells in innate immunity. Throughout the review, we will focus on new findings in innate immunity as well as features that are unique to the lung.

## Introduction

Pneumonia is a major health concern, causing significant morbidity and mortality annually, even prior to the COVID-19 pandemic [1]. Clinically, pneumonia is defined by a constellation of symptoms (cough, fever, shortness of breath), plus a new infiltrate on chest imaging [2]. Within this broad definition, the severity of illness can range from mild to severe, potentially complicated by sepsis, bacteremia, shock, acute respiratory distress syndrome (ARDS), and multiorgan system dysfunction. Pneumonia can be caused by multiple pathogens, including gram-positive and gram-negative bacteria, viruses, and fungi [3]. Despite the broad array of causative agents, there are many common pathways in the immune responses to pneumonia, suggesting host immune dysfunction as an underlying determinant of severe pneumonia.

**Fig. 1:**
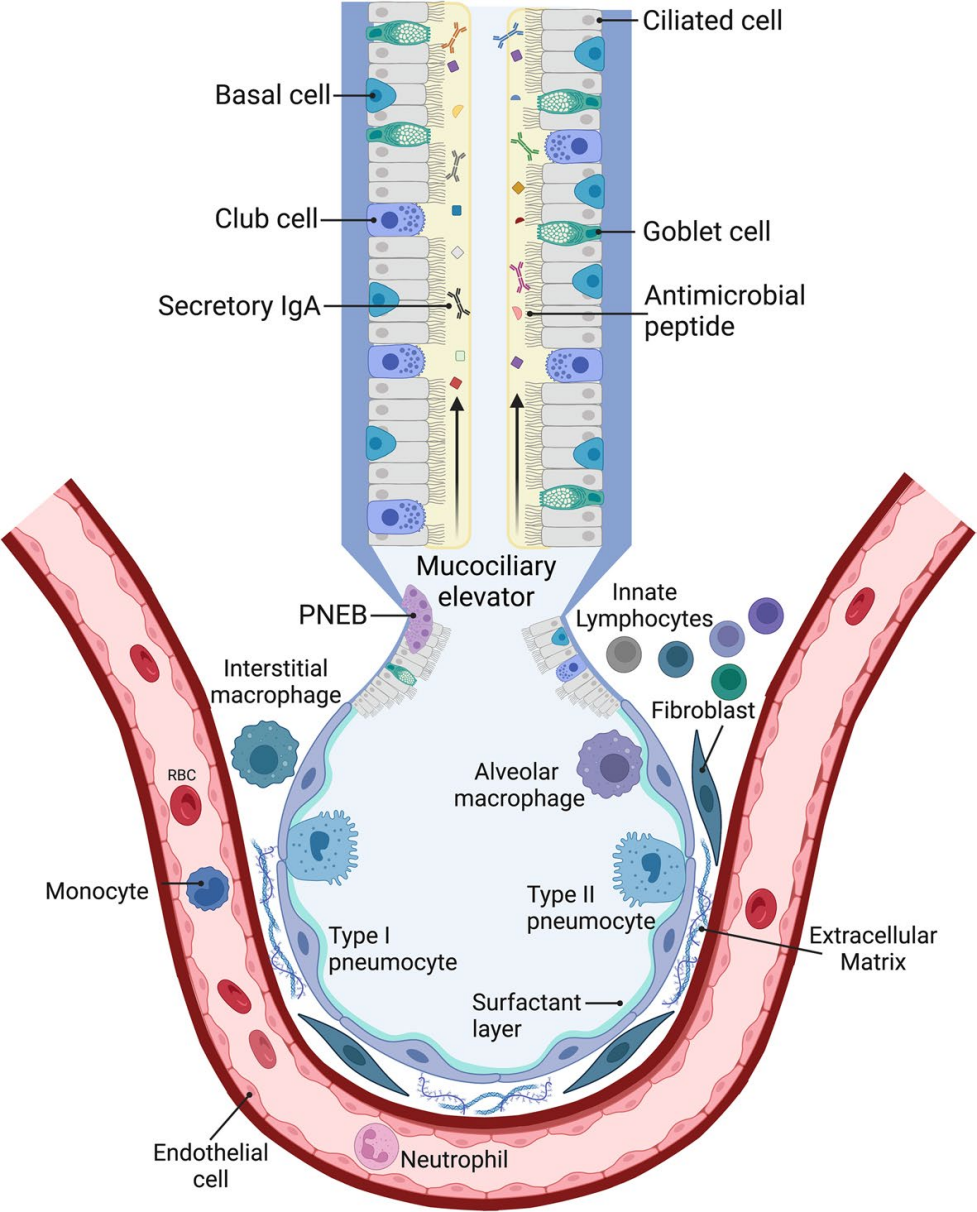
Overview of features of the innate immune response in the lungs. Multiple immune and non-immune cell types participate in the pulmonary innate immune response. Airway epithelial cells produce mucus and antimicrobial peptides, which traps and neutralizes debris and pathogens, and removes them from the lung via the mucociliary elevator. Resident immune cells, including macrophages and lymphocytes, and alveolar epithelial cells surveil the airway and alveolar spaces for potential pathogens, secreting chemokines, and cytokines to recruit additional effector cells such as neutrophils and monocytes. Migration of these cells to the alveolar space through the extracellular matrix is facilitated by vascular endothelium, fibroblasts, and alveolar epithelial cells

The innate immune system is ancient, and many aspects are conserved across vertebrates, invertebrates, and plants. In one sense, it can be defined as those aspects of host defense encoded in the germline that do not require the gene rearrangement central to adaptive immunity. Functionally, members of the innate immune response recognize conserved molecular patterns, raise immediate defensive mechanisms, alert and recruit other members of the immune response to the site of infection, and coordinate responses with the adaptive immune response. This system includes physical barriers, pattern recognition mechanisms, cell killing by immune cells and antimicrobial proteins, and coordination via cytokine signaling (Fig. 1). In this review, we will describe the features of the innate immune response, particularly how it relates to the lung and its role in pneumonia.

## Host defense at initial contact

### Anatomical barriers

The average human lung inspires approximately 11,000 L of air daily, containing a mixture of gases, fine particulates (e.g., pollen and debris) and microorganisms. As such, the lung has several anatomical barriers which constitute the first line of defense against lung pathogens. The epithelial cells of the conducting airways form a continuous physical and chemical barrier of pseudostratified epithelium in the proximal airways with simple cuboidal epithelium in more distal bronchioles. While their function is heterogenous, all epithelial cells of the lung share in their ability to regulate paracellular permeability through tight and adherens junctions. These proteins, reviewed extensively elsewhere [4], provide a physical barrier between the airspace and interstitium through their connections with neighboring cell cytoskeletal proteins. These paracellular interactions prevent dissemination of microorganisms and vascular leak, which may be disrupted during inflammatory injury by host factors such as TNF-α (tumor necrosis factor alpha) and pathogen-derived toxins, such as pneumolysin and lipopolysaccharide [5, 6].

The coordinate action of ciliated and goblet cells of the upper airways also provides a requisite function for anatomical defense: mucociliary clearance. The upper airway is lined with a complex airway surface liquid, comprised of a gel-like upper layer made of mucus that physically traps microbes and particles from the environment and a more fluid-like lower layer that facilitates the beating of cilia. While containing more than 200 proteins, the mucus layer is primarily made up of mucins, particularly MUC5AC and MUC5B (mucin 5AC and 5B), secreted by goblet cells and submucosal glands. Recently reviewed in detail [7], these polymeric glycoproteins assist in the physical composition of the airway lining fluid that promotes mucociliary clearance of > 90% of inhaled particles. Moreover, mucins are known to have direct antimicrobial function. MUC5B is especially critical for homeostatic regulation of mucociliary clearance, the lack of which leads to chronic infection and a reduction in macrophage function and interleukin-23 (IL-23) production [8]. Meanwhile, MUC5AC is important in host defense against influenza virus, potentially through its interactions with α2,3-linked sialic acid [9]. Interestingly, MUC1 (mucin 1), a transmembrane mucin expressed by both epithelial and immune cells, has an immunomodulatory role to dampen inflammatory signaling during respiratory infection through direct interaction with toll-like receptors (TLRs) [10–13]. Increased mucin secretion after either influenza or *Streptococcus pneumoniae* infection was recently shown to be partially dependent on type I interferon signaling, the production of which was also associated with in increased microbial shedding and transmission [14].

### Antimicrobial proteins

In addition to mucins, airway lining fluid contains additional proteins that aid in the immediate defense against potential pathogens. Among these proteins are immunoglobulins generated through adaptive immune pathways, which we have included in this section for completeness, but require previous infection to activate a specific memory response. Subepithelial plasma cells produce secretory immunoglobulin A (s-IgA), which are IgA dimers covalently connected to a glycoprotein called the secretory component. Both s-IgA and the secretory component are transcytosed into the airway lumen by a polymeric immunoglobulin G (IgG) receptor located on lung epithelial cells, providing memory protection against influenza and *S. pneumoniae* in an antigen specific manner [15–18]. Interestingly, the secretory component and s-IgA, together or alone, can ward off infection in a non-specific fashion as well. This occurs through interaction with bacterial components, or through immunomodulation of host factors, such as inhibition of interleukin-8 (IL-8). Lactoferrin and lysozyme are two additional components of epithelial lining fluid in the lungs [19]. Lactoferrin, in addition to iron sequestration, has been shown to disrupt microbial biofilms produced by *S. pneumoniae*, an important colonizer of the nasopharynx and causative agent of community-acquired pneumonia [20]. Lysozyme, which enzymatically cleaves the peptidoglycan backbone of bacteria, directly contributes to immune defense in the lung against both gram-positive and gram-negative pathogens [21, 22]. In addition to those mentioned above, other humoral factors that contribute to rapid defense against lung infection include complement and other acute phase proteins, such as C-reactive protein, serum amyloid proteins and pentraxin 3 [23].

A unique and critical component of the alveolar milieu is pulmonary surfactant, secreted by type II alveolar epithelial cells. Vital to reducing surface tension in the lung, surfactant proteins A and D (SP-A and SP-D) are collectins that facilitate the clearance of both viral and bacterial pathogens through enhanced uptake, agglutination, or interference with their cognate receptor, particularly in the case of viral infection [24]. Recently, it has been shown that both SP-A and SP-D are targets of neutrophil elastase, a result that may partially explain the increased susceptibility of patients with alpha-1 antitrypsin deficiency to bacterial lung infections [25]. Surfactant proteins also modulate inflammation through their interactions with both innate and adaptive cells in the lung, the details of which have been reviewed elsewhere [24]. Finally, serum SP-D has been identified as a strong predictor of severity of community-acquired pneumonia in children, likely resulting from increased alveolar permeability commonly seen during severe lung infection [26]. Other collectins, such as mannose-binding lectin, ficolins-1–3 and collectins-10–11, recently reviewed in detail [27], recognize carbohydrate moieties and provide rapid protection from a myriad of respiratory pathogens, largely through complement-mediated opsonization.

## Pattern recognition receptors & signaling pathways

A key factor of the innate immune response is very rapid (minutes to hours) response to pathogens or tissue injury. Recently reviewed elsewhere [28, 29], this is facilitated by the binding of molecules containing pathogen-associated molecular patterns (PAMPs) or damage-associated molecular patterns (DAMPs) to their cognate pattern recognition receptors (PRRs) on the surface of host cells. In general, PAMPs are conserved molecular motifs expressed by microorganisms but not host tissues, while DAMPs are host-derived molecular motifs that are only released during tissue injury. Binding of PAMPs/DAMPs to PRRs leads to the activation of innate immune signaling pathways, production of inflammatory cytokines and chemokines and the recruitment of effector cells. In this section we will describe the PRRs and their main downstream signaling pathways.

### Toll-like receptors

The first and most well-characterized of the pattern recognition receptors are toll-like receptors (TLRs, Table 1), which are a group of transmembrane proteins located either on the plasma or endosomal membrane. Their ectodomains contain leucine-rich repeats that are also their primary ligand binding domains. Ligands are conserved, vary amongst receptors, and include microbial products such as lipopolysaccharide (LPS, binds TLR4), microbial membrane lipids (lipotechoic acid, lipoarabinomannan or zymosan, binds TLR1/2 and TLR2/6 heterodimers), flagellin (binds TLR5) and unmethylated CpG DNA (binds TLR9). TLR binding and signaling has been reviewed in detail recently [28], thus the focus here will be on recent advances during lung infection. Administration of a cocktail of TLR agonists (Pam2-ODN, TLR2/6 and 9 agonists) during *Sendai paramyxovirus* infection leads to alleviation of lung epithelial damage early in infection, reduced viral burden and decreased mouse mortality [30]. In an observational study, elderly patients with severe pneumonia exhibited lower levels of monocyte TLR2 and TLR4 expression, correlating with diminished serum concentrations of interleukin-1 (IL-1), interleukin-6 (IL-6), and TNF-α [31]. Moreover, patients, especially children, with a deficiency in MyD88, are much more susceptible to pneumonia, likely due to defects in TLR signaling [32]. During *S. pneumoniae* pneumonia, knockout of TIR8 (Toll/interleukin-1 receptor 8), which is a negative regulator of TLR signaling, improves bacterial clearance and survival due to increased phagocytosis by leukocytes in the lung [33]. TLR agonists are even being considered as treatments against pneumonia, as TLR2/6 and TLR9 agonists have been effective at reducing mortality when paired with oseltamivir during influenza A infection in mice [34]. In addition, the combination of TLR5 agonists and antibiotics improves infection outcome even in response to antibiotic-resistant *S. pneumoniae* [35]. Conversely, TLR signaling may be detrimental to pneumonia outcome, particularly when immunopathology is the primary contributor to disease, as has been shown for TLR3 following infection with *Klebsiella pneumoniae* [36].

**Table 1:** Toll-like receptors (TLR)

Name	Ligand	Location	Function	Downstream signaling	Ref
TLR1	Bacterial lipoproteins	Plasma membrane	Inflammatory cytokine	TIRAP/MAL, MyD88	[28]
TLR 2	Bacterial lipoproteins	Plasma membrane	Inflammatory cytokine	TIRAP/MAL, MyD88	[28]
TLR 3	dsRNA	Endosomal membrane	IFN-1	TRAM, TRIF	[28]
TLR 4	LPS	Plasma membrane	IFN-1	TRAM, TRIF	[28]
TLR 5	flagellin	Plasma membrane	Inflammatory cytokine	TIRAP/MAL, MyD88	[28]
TLR 6	Bacterial lipoproteins	Plasma membrane	Inflammatory cytokine	TIRAP/MAL, MyD88	[28]
TLR 7	ssRNA	Endosomal membrane	Inflammatory cytokine	TIRAP/MAL, MyD88	[28]
TLR 8	ssRNA	Endosomal membrane	Inflammatory cytokine	TIRAP/MAL, MyD88	[28]
TLR 9	CpG DNA	Endosomal membrane	Inflammatory cytokine	TIRAP/MAL, MyD88	[28]

### NOD-like receptors

NOD-like receptors (nucleotide-binding oligomerization domain-like receptor, NLRs Table 2) are a diverse set of intracellular receptors that respond to a wide variety of PAMPs and DAMPs. While they share conserved structural motifs, their mechanisms of activation and downstream function are quite varied. Some NLRs, such as NLRP3, have oligomerization capabilities to form inflammasomes that lead to the activation of cell death pathways and the proteolytic cleavage and release of interleukin-18 (IL-18) and interleukin-1β (IL-1β). In addition, other products released by dead and dying cells, called alarmins, include HMGB1, IL-33 and IL-1α. Alarmins promote host defense and inflammation through a variety of mechanisms. HMGB1 interacts with its receptor RAGE, which is exclusively in high concentrations in the lung and promotes pro-inflammatory signaling through NF-kB activation [37]. IL-33 and IL-1α are both IL-1 family cytokines, signaling through their receptors ST2 and IL-1 receptor, respectively. IL-33 provides protection against pneumonia-induced acute lung injury, potentially mediated through increased eosinophilia and lower neutrophil recruitment [38], while the role of IL-1α (in comparison to IL-1β) are poorly understood, but likely act through liver-dependent mechanisms [39].

**Table 2:** NOD-like receptors (NLR)^a^

Subfamily	Select subfamily member	Ligand	Function	Signaling	Ref
NLRA	CIITA	IFNγ	Positive regulator of MHCII transcription	Transcription factor for MHC	[40]
NLRB	NAIP	Flagellin; T3SS	Anti-apoptosis	Caspase-1 mediated inflammasome	[41]
NLRC	NOD1	iE-DAP dipeptide	Inflammatory cytokine; antimicrobial proteins	RIP2, NFκB, MAPK	[42]
	NOD2	Muramyl dipeptide (MDP)	Inflammatory cytokine; antimicrobial proteins	RIP2, NFκB, MAPK	[43]
	NLRC3	Viral DNA & nucleic acids	Neg reg of NFκB; attenuates type I IFN	TRAF6	[44]
	NLRC4	Flagellin; T3SS; T4SS	Inflammasome formation	Caspase-1	[45]
	NLRC5	Nucleoside triophospate	Regulation of MHCI	MHCI transcription	[46]
	NLRX1	dsRNA, PUA, ESA, and DHA	Negatively regulates IFN-I and IL-6 responses; located on mitochondria	NFκB	[47]
NLRP	NLRP1	MDP, anthrax lethal toxin	Inflammasome formation	Caspase-1	[48]
	NLRP3	PAMPS and DAMPs associated with viral infection	Inflammasome formation	Caspase-1	[49]
	NLRP6	unknown	Poorly characterized inflammasome formation	Negative regulation of NFκB and MAPK	[50]
	NLRP7	lipopeptide	Putative inflammasome formation	unknown	[51]
	NLRP12	unknown	Unproven inflammasome formation	Negative regulation of NFκB and MAPK	[52]

^a^Select receptors with established role in innate immunity

Non-inflammasome generating NLRs (NLRC1-3, NLRC5 and NLRX1) have several important roles during infection, mediating both immune defense (pro-inflammatory) and resolving (anti-inflammatory) pathways. The details surrounding NLR activation and signaling were recently reviewed elsewhere [48, 52]. Expression of NLRC4 is increased in both human and mouse lungs with pneumonia. Interestingly, during experimental pneumonia with *Staphylococcus aureus,* mice exhibit increased survival and reduced bacterial burden with NLRC4 ablation. Protective effects of NLRC4 deficiency were shown to occur through the inhibition of necroptosis and improvement in interleukin-17A (IL-17A)-dependent neutrophil recruitment [53]. Similarly, activation of the NLRP6 inflammasome impeded neutrophil influx and antimicrobial activity during *S. aureus* infection, likely through the increase in inflammatory cell death pathways (pyroptosis and necroptosis) and reduction in interferon-γ (IFN-γ) [50]. However, limiting NLRP6 signaling is not always beneficial. NLRP6 deficiency during *K. pneumoniae* infection results in increased mortality and extrapulmonary bacterial dissemination due to a defect in neutrophil recruitment and neutrophil extracellular trap (NET) formation [54]. Finally, mice deficient in NLRP3 or its associated adaptor ASC (apoptosis-associated speck-like protein containing a CARD) demonstrated a robust, early production of cytokines, leading to improved clearance of *S. pneumoniae* serotype 3 [55]. Whether NLRs promote a response that facilitates bacterial clearance appears to be context specific, depending on pathogen, strain, and/or pathogen burden.

### RIG-I-like receptors

RIG-I-like receptors (retinoic acid-inducible gene-I like, RLR, Table 3) are intracellular sensing molecules that respond to viral RNA and mediate the production of type I and type III interferons. There are three known RLRs (RIG-I, melanoma differentiation-associated protein 5 (MDA5), and laboratory of genetics and physiology 2, (LGP2)), each having a conserved C-terminal domain that recognizes RNA and a central helicase domain. In addition, RIG-I and MDA5 contain N-terminal caspase recruitment domains (CARDs) which facilitate their interaction with an adaptor protein, MAVS (mitochondrial antiviral signaling protein). LGP2, lacking the N-terminal CARD, does not interact with MAVS, nor is it thought to act as a signaling molecule [56]. During respiratory syncytial virus (RSV) infection, alveolar macrophages produce type I interferons through activation of the RIG-I-MAVS complex, leading to enhanced inflammatory monocyte recruitment, limiting viral replication [57]. Interestingly, severe acute respiratory syndrome coronavirus (SARS-CoV) and the pandemic SARS-CoV2 have been shown to interfere with RIG-I activation to limit type I interferon production, therefore, increasing viral replication [58, 59]. Recent work has shown that RIG-I limits SARS-CoV2 replication in lung cells through a non-canonical, MAVS-independent, mechanism that is dependent on the helicase domain of RIG-I [60]. While its biological relevance needs to be confirmed in vivo, this may represent a unique mechanism for boosting immunity towards coronavirus infection. Moreover, SARS-CoV2 nucleoprotein interferes with MAVS activation by RIG-I through its helicase domain, preventing type I interferon activation. Viral interference was inhibited by a peptide that targets the dimerization domain of the viral nucleoprotein and prevented severe lung damage and limited viral replication in humanized transgenic mice infected with SARS-CoV2. These new findings suggest that RLR signaling is critical to limiting coronavirus infection, the mechanisms of which are likely relevant to other RNA viruses as well [61]. Finally, while RLR-mediated host defense has been largely attributed to viral infection, studies have shown that *Legionella pneumophila*, the causative agent of Legionnaire’s disease can activate the RIG-I/MDA5 pathway due to its ability to inject genetic material into the cytosol via a type-IV secretion system [62]. Future studies should consider the role of RLR signaling in other intracellular bacterial species.

**Table 3:** RIG-I-like receptors (RLR)

Name	Ligand	Location	Function	Signaling	Comment	Ref
RIG-I	Viral RNA	Cytoplasm	Interferon I and III production	MAVS		[56]
MDA5	Viral RNA	Cytoplasm	Interferon I and III production	MAVS		[56]
LGP2	Viral RNA	Cytoplasm	Unclear antiviral effect	None	Possible negative regulator of RIG-I and MDA5	[56]

### DNA-sensing receptors

In addition to pattern recognition receptors that recognize RNA, there are DNA-sensing receptors (Table 4), such as AIM2-like receptors (absent in melanoma 2-like, ALR), cyclic GMP-AMP synthase (cGAS), and interferon gamma inducible protein 16 (IFI16). Most of these receptors use STING (stimulator of interferon genes) as an adaptor protein [63]. The cGAS-STING pattern recognition pathway is an intracellular DNA-sensing system that results in the activation of type I interferon. In the lungs, cGAS has been reported to recognize gram-positive and gram-negative bacteria, such as *S. pneumoniae* and *Pseudomonas aeruginosa*, and mediates protection against pneumonia caused by *P. aeruginosa* infection [64]. Whole-body STING^−/−^ mice have increased inflammatory cytokine levels, alveolar edema, macrophage necroptosis, and an increase in bacterial burden during *S. aureus* infection [65]. Interferon-inducible protein 204 (IFI204; the murine homolog of IFI16) is an AIM2-like, intracellular DNA sensor, reported to facilitate type I interferon responses to intracellular bacterial infection through cGAS, STING and interferon regulatory factor 3 (IRF3) [66, 67]. More recently, it has been shown that IFI204 is protective against extracellular *S. aureus* infection of the lungs in a way that is independent of type I interferon induction. IFI204 ablation in mice led to decreased survival and increased bacterial burden and lung injury in conjunction with a reduction in macrophage and neutrophil extracellular traps [68]. Interestingly, AIM2-like receptors, have been shown to be dispensable for type I interferon production, indicating they may have an alternate function [69].

**Table 4:** DNA-sensing receptors

Name	Ligand	Location	Function	Signaling	Ref
cGAS	Cytosolic DNA	Cytoplasm	Production of type I IFN and pro-inflammatory cytokines	STING	[70]
IFI16/IFI204	Intracellular ss and dsDNA	Nucleus	Type I IFN	STING	[71]

### C-type lectin receptors

C-type lectin receptors are a heterogenous superfamily of receptors, comprising of more than 1000 proteins, subdivided amongst 17 smaller groups. They perform a wide variety of biological functions, including but not limited to regulation of apoptosis, growth and development, and antimicrobial immunity. In reference to the latter, many C-type lectin receptors, such as the collectins and dectin-1, serve as pattern recognition receptors (Table 5, [72]). The majority of C-type lectin receptors remain uncharacterized in their roles against respiratory pathogens, however, C-type lectin domain family 4 member D (CLEC4D), is protective against *K. pneumoniae-*induced pneumonia, whereby Clec4d knockout mice exhibit increased mortality, lung damage and bacterial CFU, which may be attributed to a defect in neutrophil clearance [73]. Dectin-2, which binds to the fungal pathogen *Pneumocystis jirovecii*, activates pro-inflammatory cytokine signaling in response to fungal infection in the lung; however, dectin-2 deficiency does not appear to have an impact on fungal burden [74]. Among the C-type lectin receptors that respond to respiratory infection, such as versican and surfactant protein D, Mincle (macrophage inducible C-type lectin receptor) has been widely studied and shown to have prominent role against pneumonia. In response to *K. pneumoniae* infection, Mincle deficiency led to a progressive increase in bacterial burden, despite an exaggerated inflammatory response which promoted severe lung damage. Moreover, Mincle-insufficient mice showed defects in neutrophil phagocytosis and NET formation which may partially explain their inability to control infection [75]. Mincle also recognizes *S. pneumoniae*, however its role in response to pneumococcal infection is inconsistent in the literature. In one study, Mincle-knockout mice were unable to clear bacteria from the lungs, which was accompanied by an enhanced inflammatory response. However, in contrast to *K. pneumoniae* infection, they did not display deficiencies in neutrophil phagocytosis [76]. A second study showed that Mincle knockout mice exhibited no changes in inflammatory responses, phagocytosis or bacterial killing in response to *S. pneumoniae* infection [77]. Finally, a more recent study showed that transgenic overexpression of Mincle in mice led to activation of the NLRP3 inflammasome, leading to increased lung injury and fatality in response to pneumococcal pneumonia, which could be abrogated through NLRP3 inflammasome inhibition [78]. Mincle-deficient mice also exhibit increased fungal burden in response to *P. jirovecii* infection, however with no change in mortality, which may be attributed to an increase in the anti-inflammatory protein, IL1RA [79]. In sum, many more C-type lectin receptors likely influence lung infection but remain unexplored. For example, lectin-like oxidized low density lipoprotein receptor-1 (LOX-1), has been shown to mitigate sepsis-induced lung injury in models where it is systemically inhibited [80, 81], but its contribution to the local response in the lung is not yet known. These and others should be the subject of future research.

**Table 5:** C-type lectin receptors^a^

Name	Ligand	Location	Group	Signaling	Ref
CLEC4D (MCL)	α-mannans	Cell surface	II – Type 2 receptors	NF-κB	[73]
Surfactant Protein A&D	Respiratory pathogens	Secreted	III—Collectins	Enhancement of phagocytosis & IL4/IL13 repair pathway	[82]
Dectin-1	β-glucan; galectin-9; annexins; vimentin; tropomyosin; N-glycan	Cell membrane	V – NK receptors	Syk-dependent ROS; NFAT and NFκB signaling	[83]
Dectin-2	α-mannans	Cell membrane	II – Type 2 receptors	Syk, PKCδ and CARD9–Bcl10–Malt1	[84]
Versican	CD44, PSGL-1, TLR2; P and L-selectins	Extracellular	I—Proteoglycan	MyD88	[85]
Mincle	Glucosyl-diacylglycerol (Gly-DAG); microbial lipidic species	Cell surface	II – Type 2 receptors	Syk, PKCδ, CARD9–Bcl10–Malt1 and MAPK	[86]

^a^Selection of receptors discussed in text

### Scavenger receptors

Scavenger receptors are a highly diverse set of cellular receptors that have been subdivided into ten classes based on their structural characteristics and biological function (Table 6). One commonality amongst all scavenger receptors is that they bind to a heterogenous group of ligands, including but not limited to, modified lipoproteins, microbial components (LPS, lipoteichoic acid (LTA), and CpG), apoptotic cells and advanced glycation end products. C-reactive protein (CRP) is another known ligand of some scavenger receptors, including LOX-1 and SR-A [87, 88], and plays an important role in host defense against bacteria through complement-mediated opsonization [89]. Complement fixation, a highly conserved mechanism of immune defense and regulation through its recognition of microbial and damage associated molecular patterns is a critical contributor to host defense and rapid response to bacterial infection, the details of which have been reviewed elsewhere [90, 91]. In addition to ligand binding, some scavenger receptors, such as SR-A, LOX-1, P2X7, SSc5D, have also been shown to bind bacteria directly, which contributes to their uptake and killing [92–95]. Scavenger receptors have diverse roles in health and disease, displaying both pro- and anti-inflammatory roles depending on the receptor’s location, ligand and ability to interact with co-receptors, such as TLR4 [96]. During pneumonia, there is a paucity of recent data on the role of most scavenger receptors, although older studies have shown prominent roles for MARCO (macrophage receptor with collagenous structure) [97–99], CD36 [100], and SR-A (scavenger receptor-A) [101, 102] in response to lung infection. However, recently it was shown that CD5L (CD5 molecule-like), which is a scavenger receptor in the cysteine-rich superfamily, contributes to a more severe murine response to methicillin resistant *S. aureus* (MRSA) in the lung. Antibody-mediated inhibition of CD5L led to decreased mortality, along with dampened lung cytokine levels, and decreased bacterial burden, while CD5L supplementation increased mortality, lung injury lung cytokines and bacterial burden. Interestingly, addition of CD5L also increased phagocytic uptake of MRSA in macrophages and neutrophils but had no effect on bacterial killing [103]. Alternatively, the scavenger receptor CD36, promotes phagocytosis and killing of *K. pneumoniae* in a capsule-independent manner. CD36-deficient mice also have a reduced cytokine profile and impaired phagocytosis, likely the result of lower sensitivity to LPS [104]. Finally, SR-BI (scavenger receptor class B type I), a scavenger receptor that mediates uptake of cholesterol ester, is also important for lung defense against *K. pneumoniae*. SR-BI-deficient mice have a very similar phenotype to those lacking CD36, whereby mortality, lung injury, inflammatory cytokine levels and bacterial replication and dissemination are increased. Additionally, more neutrophils are recruited to the lung in a corticosterone-dependent manner and like with the previously mentioned scavenger receptors, phagocytosis is impaired in mice lacking SR-BI. Finally, it was revealed that SR-BI is necessary for efficient clearance of LPS, without which LPS signaling is prolonged [105]. Taken together, the current data suggest that scavenger receptors play an important role in the defense against lung pathogens, and considerable work is required to elucidate their specific, microbe-dependent functions.

**Table 6:** Scavenger receptors^a^

Name	Ligand	Location	Group	Signaling	Comment	Ref
LOX-1	Oxidized lipoproteins; activated platelets; advanced glycation end products; apoptotic cells; live bacteria	Cell Surface	Class E	NFκB; MAPK; NADPH oxidase	Also a C-type lectin receptor	[106]
MARCO	Bacteria; LPS; LTA; CpG DNA	Cell surface	Class A	Unclear, cooperation with TLRs		[96]
CD36	Long chain fatty acids; PAMPs; DAMPs; proteins containing TSR domains	Cell Surface	Class B	MAPK; PPAR-γ; NF-κB; ROS		[107]
SR-A	HSPs; PAMPs; oxidized LDL; proteoglycans; poly I:C; major vault protein	Cell surface	Class A	PI3K; NF-κB; MAPK		[108]
CD5L	CD36; PAMPs; Live bacteria	Secreted	Class I	Inhibition of apoptosis	Unknown mechanism	[109]
SR-BI	HDL; modified LDL; phospholipids	Cell surface	Class B	eNOS activation; phospholipase C & D; PI3K; MAPK		[110]

^a^Selection of receptors discussed in text

## Leukocyte-driven defense

Leukocytes participate in the innate immune response by acting via sentinel and/or effector functions. Sentinel cells are resident in the in local tissue and rapidly respond to the presence of PAMPs and DAMPs in the environment. Effector cells are generally circulating and are recruited to the site of infection or injury by signals produced by sentinel cells. Some innate immune cells can act in both a sentinel and effector cell capacity, such as alveolar macrophages and innate lymphocytes. In this section, we will discuss the leukocytes that participate in the innate immune response.

### Alveolar macrophages

Alveolar macrophages (AMs) are the most well-characterized resident immune cells in the lung. As their name suggests, they are located in the alveolar compartment, where they constitute the first line of defense against invading microorganisms through their high phagocytic capacity. Additionally, they promote tissue repair and lung homeostasis. AMs are long-lived cells that are closely positioned to type II alveolar epithelial cells, where they are responsible for ingesting and breaking down pulmonary surfactant and fostering tissue homeostasis even in the wake of injury [111, 112]. Epithelial-derived GM-CSF (granulocyte–macrophage colony-stimulating factor) promotes the establishment and differentiation of AMs through activation of PPAR-γ (peroxisome proliferator-activated receptor γ) the lack of which results in defective AM development and pulmonary alveolar proteinosis due to surfactant buildup [113]. The initial pool of AMs are derived from yolk sac precursors of fetal monocytes, but are gradually replaced by monocyte-derived macrophages throughout the life of the host [114]. Differential function of fetal derived versus monocyte-derived macrophages is the subject of active investigation, reviewed in [115]. Low-level intrusions into the alveoli are quickly and efficiently cleared by AMs through the production of antimicrobial proteins, reactive oxygen species and phagocytic killing. Once invading microorganisms reach a certain threshold beyond the killing capacity of AMs, they will secrete cytokines, chemokines and arachidonic metabolites to recruit neutrophils that will aid in clearance of pathogens [116]. Both processes will be discussed in greater detail in the following sections.

#### Pathogen recognition and killing

Like most immune and non-immune cells, AMs recognize microorganisms through their expression of PRRs which bind to conserved motifs found on different classes of microbes. This interaction and subsequent signaling alerts the macrophage to the threat, allowing for direct microbial killing by phagocytic uptake, nitric oxide, hydrogen peroxide and reactive oxygen species generation [117, 118]. Recognition of flagellated *P. aeruginosa* was shown to depend on macrophage expression of TLR5, which promoted IL-1β-dependent phagocytosis and killing of the pathogen through production of asparagine endopeptidase [119]. In contrast, phagocytic capacity of AMs is dampened during secondary pneumonia due to an altered microenvironment elicited by a primary infection with *Escherichia coli* or *S. aureus*, leading to defective clearance of bacteria*.* Although not yet identified, alveolar components in recovered mice increase SIRPα (signal regulatory protein α) expression in AMs, which inhibits their phagocytic capacity through regulation of both cellular metabolism and gene transcription [120]. Interestingly, bacterial clearance improves following repeated exposures of mice to differing strains of *S. pneumoniae*, leading to transcriptional and metabolic reprogramming of AMs [121]. Notably, in humans, experimental colonization of the nasopharynx with *S. pneumoniae* (serotype 6B) increased the homo- and heterotypic opsonophagocytic capacity of AMs. Activation of AMs correlated with microaspiration of pneumococcus into alveolar fluid and depended on type 1 helper (Th1) T-cells differentiation and the resulting low levels of IFN-γ, which promoted the production of TNF-α by AMs and increased the differentiation of monocytes to macrophages [122]. Age-related deficiencies in AM proliferation were recently found to be dependent on the alveolar microenvironment. Increased levels of hyaluronan in aged mice confers a hypo-responsive phenotype to GM-CSF in AMs, reducing their proliferative capacity and increasing the severity of influenza A infection [123]. This confirmed a previous report that found aging compromises AM numbers and function [124].

Interferon-gamma receptor (IFNGR1) signaling on AMs renders contradicting outcomes depending on the etiology of lower respiratory infection. IFN-γ promotes clearance of RSV in neonatal mice in a manner that is dependent on lung macrophages, with AMs exhibiting increased expression of MHCII (major histocompatibility complex II), a direct indication of their activation [125]. However, bacterial clearance with a non-lethal strain of *S. pneumoniae* following influenza is impaired and becomes fatal through IFNGR1 signaling on AMs and other mononuclear phagocytes [126]. Type I interferon similarly exacerbates severity of secondary bacterial pneumonia with MRSA, potentially through the activation of macrophage STAT2 (signal transducer and activator of transcription 2) [125]. Abrogating this response improves bacterial clearance due to the presence of macrophages expressing both inflammatory and regulatory surface markers (M1/M2 macrophages), which represent pro-inflammatory versus pro-resolving macrophages, respectively [127]. Infection of mice with RSV induced an M2-like (regulatory) phenotype in AMs that is dependent on an increase in Gas6/Axl (growth arrest-specific protein 6) signaling, typically associated with efferocytosis. Polarization of AMs resulted in increased susceptibility to secondary pneumococcal infection due to lower production of IL-18 from AMs and IFN-γ from natural killer cells [128]. Future studies should aim to identify critical alveolar components and signaling mechanisms that give rise to long-lived alterations in AM function and to characterize pathogen-specific differences in aforementioned phenotypic changes. While resident AMs are often sufficient to eradicate smaller and/or less virulent pathogenic challenges and maintain alveolar tissue integrity [129], higher levels of pathogen burden require additional help from recruited immune cells. In addition to direct killing, AMs produce chemokines and cytokines, such as IL-8, CXCL1, CXCL2 (C-X-C motif chemokine ligand 1 and 2), IL-6 and TNF-α, to activate and recruit additional innate and adaptive immune cells.

#### Roles in neutrophil recruitment and activation

While AMs activate both the innate and adaptive arms of the immune system, the scope of the current review is on initial acute responses to infection and thus, will be focused on the recruitment of cells derived from myeloid progenitor cells. As noted previously, inflammatory insults can lead to AM dysfunction and downstream defects in neutrophil recruitment and increased bacterial burden during secondary infection. In fact, pathogens producing pore-forming toxins may directly target AMs, underscoring their central role in modulation of downstream responses [114]. Following sepsis by cecal ligation and puncture, AMs reduce their production of CXCL1 and phagocytic uptake of bacteria in a TNF-α/interleukin-10 (IL-10) mediated manner. However, this defect can be reversed with the administration of interferon-β (IFN-β) [130], the production of which may be dampened by increased levels of transforming growth factor-β1 (TGF-β1) following sepsis-mediated lung injury [131]. CXCL1 production and early neutrophil recruitment is also impaired in mice that lack macrophage FABP4 (fatty acid binding protein-4), leading to increased bacterial replication and mortality following *P. aeruginosa* infection [132]. Curiously, AM expression of TLR3 is detrimental to host response to *K. pneumoniae*, whereby TLR3^−/−^ macrophages exhibit increased phagocytic and efferocytic capacity along with a more M2-like phenotype [36]. TLR3^−/−^ macrophage responsiveness is also enhanced in that they express higher levels of CXCL1 and CXCL2, leading to greater neutrophil numbers, but interestingly exhibit diminished lung injury. This phenotype is likely due to the concordant effects of reduced bacterial burden and increased efferocytosis of dying neutrophils [36]. During viral infection, AMs produce robust levels of IL-1β through activation of the NLRP3 inflammasome [133]. Intriguingly, NLRP3 inflammasome activation, but not expression is dependent on the neutrophil-derived peptide cathelicidin-related antimicrobial peptide (mCRAMP) [133]. Thus, during viral, but not bacterial infection, IL-1β secretion requires a two-hit system on AMs through initial infection and crosstalk with recruited neutrophils [133]. Production of IL-1β during influenza/pneumococcus co-infection prevents AMs apoptosis, a process that is protective against *S. pneumoniae* serotype 14, but not 19, outgrowth. Interestingly, during co-infection in mice deficient in IL-1 receptor type 1 (Il1r1^−/−^ mice), the lack of IL-1β signaling is compensated for by TNF-α activation of recruited neutrophils [134]. TNF-α, produced by neutrophils, similarly protects against *L. pneumophila* infection through its actions on AMs, whereby TNFα signaling increases lysosomal acidification in a caspase-dependent manner [135]. AMs temper neutrophil activity during infection with *Acinetobacter baumannii,* where both neutrophil recruitment and neutrophil production of reactive oxygen species is enhanced with macrophage depletion. This is detrimental to the *A. baumannii*-infected host, who exhibit markedly enhanced lung injury and bacterial load [136]. In sum, AMs are critical determinants in the recruitment of neutrophils during infection of the alveolar compartment, the result of which may improve or worsen outcome based on the source of infection.

#### Alternative mechanisms of AM response to pneumonia

In addition to direct killing and recruitment of leukocytes to the lungs, AMs have developed alternative methods to aid in host defense and recovery from pneumonia. One mechanism is the apoptosis of the macrophage itself to prevent microbial replication [117] and has been shown to occur following infection with influenza, pneumococcus and tuberculosis, among other important pathogens [137–139]. This response was recently shown to be prevented by macrophage over-expression of the antiapoptotic protein MCL-1 (myeloid cell leukemia 1) which impaired the clearance of *S. pneumoniae* and *Haemophilus influenzae.* Importantly, macrophage apoptosis was not shown to occur following infection with *S. aureus,* indicating important pathogen-specific differences in cellular response to infection [140].

Finally, AMs are critical to tissue repair following pneumonia-induced lung injury. While beyond the scope of this review, a few studies are worth noting as avenues for further discovery. Production of the tissue-resolving growth factor, amphiregulin, is induced by AMs during *Nippostrongylus brasiliensis* infection and promotes the restoration of the blood-lung barrier. This occurs through the “inside-out” activation of integrin-α_V_ on pericytes leading to their release of TGF-β and differentiation into myofibroblasts [141]. Reparative properties of AMs are also regulated metabolically. Activation of the Wnt-β catenin signaling pathway results in increased macrophage hypoxia inducible factor 1α (HIF-1α) expression and higher glycolytic versus mitochondrial metabolism. Tipping of the metabolic scale towards glycolysis led to elevated inflammatory signaling during influenza infection at the expense of proliferation and repair [142].

### Interstitial macrophages

While AMs reside in the alveolar lumen, at a ratio of approximately one macrophage to three alveoli, interstitial macrophages (IMs) inhabit the interstitial space between the epithelium and underlying capillaries. It was thought that they reside exclusively near the bronchi [143], however recent reports have shown the existence of a CD206^−^ IM population located within the alveolar interstitium [144], and others that have shown they can traverse into the luminal space when required [145]. IMs have been categorized into three separate subtypes (IM1-3) based on expression of various markers, including CD11c, MHCII, CD206, CD169 and Lyve-1 [143], although it is unclear whether these represent three phenotypically distinct subpopulations. Two recent studies have identified two unique clusters of IMs based on expression of CD206 or Lyve1/MHCII/CX3CR1 and one population that may represent a transitional state from Ly6c-low patrolling monocytes (CD64^+^CD16.2^+^) [144, 146]. Another study identified a population of embryonic-derived, nerve associated, CD169^+^ IMs [147], which may be the same as the Lyve1^low^/MHCII^high^ population identified in the work by Chakarov et al. [146]. Ontologically, IMs have a mixed background, arising from both blood and lung-resident monocytes and yolk sac precursors and this variation may contribute to their heterogeneity. The role of IMs in response to pneumonia is less characterized than the role of AMs, however, IMs exhibit profound immunoregulatory properties in their ability to produce IL-10. In response to multiple ligands, but especially CpG DNA, IMs expand and produce large quantities of IL-10 which is protective against allergic airway disease [145]. IL-10 production has been attributed to the bronchial-associated CD206^+^ IM population, along with other immunoregulatory cytokines, such as interleukin-1 receptor antagonist (IL1-Ra) and leukemia inhibitory factor (LIF) [144]. However, CD169^+^ nerve-associated IMs also produce substantial amounts of IL-10 in response to influenza infection and poly(I:C) stimulation. Depletion of this IM subset elicits profound mortality in influenza-infected mice and greater inflammatory cytokine levels in response to poly(I:C) [147]. Moreover, signals produced by endothelial cells play an important role in the expansion and regulation of IMs. Recently it has been shown that respondin-3, a WNT ligand, is produced by endothelial cells to promote the anti-inflammatory phenotype of IMs through metabolic and epigenetic modulation. Through these changes, mice were less susceptible to LPS-induced lung injury [148]. Notably, while AMs are preferentially infected by *Mycobacterium tuberculosis* and provide a means for microbial dissemination, IMs are more capable of limiting bacterial growth through mechanisms that are dependent on nuclear factor κB (NF-κB) activation and glycolytic metabolism [149].

### Monocytes and monocyte-derived macrophages

Monocytes are a recruited cell type that are continuously produced throughout life from hematopoietic progenitor cells in the bone marrow. They are a heterogenous population of cells that can give rise to monocyte-derived macrophages or monocyte-derived dendritic cells. In addition, classical (Ly6C^hi^) and patrolling monocytes (Ly6c^lo^) have been identified in mice and humans. Increased heterogeneity amongst monocyte populations continue to be identified through the use of single-cell techniques, such as single-cell RNA sequencing, although their functional differences remain to be determined [150]. Chemokine receptors, such as CCR2 (C–C motif chemokine receptor 2), play a critical role in recruiting monocytes into the lung during infection. As such, depletion of CCR2^+^ monocytes led to higher weight loss and bacterial burden in the lungs of mice infected with all five strains of *K. pneumoniae* that were tested [151]. Inflammatory monocyte killing of *K. pneumoniae* was further shown to depend on their production of TNF-α, which activates type 3 innate lymphocytes (ILC3) to produce IL-17A, promoting ROS production by monocytes [152]. Age-dependent deficiency in host defense to *S. pneumoniae* are also affected by Ly6C^hi^ monocytes, whereby continuous levels of TNF-α in the blood of old mice promote the mobilization of immature, CCR2^+^ monocytes that are less efficient at bacterial killing, emphasizing the importance of fully functioning monocytes in host defense [153]. Recently, there has been a substantial increase in studying the role of monocytes in promoting lung injury during pneumonia, due to their implied role in SARS-CoV2 infection. While reviewed in great detail elsewhere [154, 155], there is considerable evidence that suggests monocytes (and macrophages) contribute to the immunopathology in severe SARS-CoV2 infection. Similarly, monocytes from sepsis plus ARDS patients in comparison to sepsis-only patients revealed a gene signature enriched for IFN-stimulated genes and downregulation of immunomodulation genes, such as SOCS3 (suppressor of cytokine signaling 3) [156].

### Neutrophils

Neutrophils are highly specialized, short-lived effector cells of the myeloid lineage, they are the first circulating cells to be recruited to the site of infection in pneumonia, and play an important role in clearance of invading pathogens. During acute infections, neutrophils function to clear pathogenic bacteria through three main processes—phagocytosis, degranulation and NETosis. These processes have been reviewed extensively elsewhere [157–159] and briefly summarized here. Upon arrival at the site of infection, neutrophils identify pathogens by binding microbial components to PRRs, or opsonized microbes to neutrophil Fc receptors (FcR) or complement receptors (CRs) [160]. Bound microbes are phagocytosed, and bacteria-containing phagosomes are fused with specialized lysosomal vesicles containing creating a phagolysosome in which bacteria are killed via oxidative and non-oxidative pathways [161]. Microbes remaining outside the neutrophil can also be killed through neutrophil degranulation, in which pre-packaged granules are fused to the neutrophil surface, releasing their toxic contents (proteases and reactive oxygen species, ROS) into the surrounding space [162]. Finally, through carefully coordinated condensation of chromatin, attachment of cytoplasmic and granule proteins and extrusion of DNA–protein complex, neutrophils can release neutrophil extracellular traps (NETs), which transform the extracellular environment, trapping and killing nearby microbes [163]. Although critical to pathogen clearance, these neutrophil functions often underlie the significant bystander cell death and tissue injury seen in pneumonia and ARDS [164, 165]. Neutrophils also directly influence the immunological responsiveness of other cells via alteration in surface markers, cytokine production and direct cell contact [166–168]. Finally, neutrophils are transcriptionally dynamic, regulating inflammatory pathways at a transcriptional level in response to a variety of stimuli [169–172].

While there are many aspects of neutrophil biology that are broadly applicable to all organ systems, the structure of the lung has a direct impact on neutrophil biology [173–176]. The pulmonary capillary bed is composed of narrow segments, with a vessel diameter ranging from 2-15 µm, compared with the average neutrophil diameter of 6–8 µm [177], and lower pressure than systemic circulation. Because of this narrow, low-pressure system, neutrophils transit the pulmonary capillary bed slowly, leading to a large number or “marginated” pool of neutrophils in the lungs at any one time [158, 177]. In terms of neutrophil recruitment, slow transit time through the lung obviates the need for the selectin- and integrin-mediated neutrophil capture and rolling that is central to neutrophil migration out of the vasculature in other organs [175]. Selectin- and integrin-mediated signaling is not required for neutrophil migration out of the vasculature in *S. pneumoniae* pneumonia, but is required in with other pathogens [178–181]. In addition, non-canonical adhesion receptors also participate in neutrophil recruitment and migration in respiratory infections [182, 183].

Emerging evidence also suggests that neutrophil responses, particularly as they relate to cytokine production and surface marker expression, can vary based on tissue location and/or pathogen stimulus [184]. Neutrophils that have migrated to the lungs have a distinct transcriptome from circulating neutrophils after endotoxin inhalation [185] or severe RSV infection [186]. Additionally, the chemokine repertoire secreted by pulmonary neutrophils is different than circulating neutrophils in influenza [187]. Full characterization of pulmonary-neutrophil responses in infection is ongoing.

In addition to manifesting tissue-specific responses, it also appears that neutrophils in each tissue can be comprised of functionally distinct subpopulations. Recent studies employing flow cytometry and single cell sequencing have demonstrated subpopulations of neutrophils that can be maintained at baseline and during infection [188–192]. This neutrophil heterogeneity has been characterized in multiple ways, such as N1 versus N2 [193–195], high density vs low density [196–198], activated vs refractory [199], or inflammatory vs inhibitory [188]. While there is good evidence for heterogeneity of neutrophil responses, it is still not clear how universal this heterogeneity is, or whether neutrophil subpopulations are recapitulated in the lung during pneumonia.

### Innate lymphocytes

In addition to their well-known role in adaptive immunity, a subset of lymphocytes also plays an important role in innate immunity. These cells are characterized by limited or no antigen receptors and respond instead to host alarm signals and pathogen products. They respond quickly to stimulus and play a crucial role interacting with other innate effector cells (such as macrophages and neutrophils), as well as adaptive immune responses mediated by T cells, B cells, and dendritic cells. These “innate” lymphocytes are divided into T cells with limited antigen diversity (γδ T cells, iNKT cells, and MAIT cells), and lymphoid cells lacking antigen receptors, but with lymphocyte morphology (Innate Lymphoid Cells—NK cells, ILC1, ILC2, ILC3, and LTi cells). In this section, we will focus on those innate lymphocytes with a clear role in pulmonary immunology, specifically γδ T cells, iNKT cells, MAIT cells, NK cells, and ILC2 cells.

#### Innate T cells

##### γδ T cells

These cells express T cell receptor (TCR) heterodimers containing gamma and delta chains, with a limited diversity [200, 201], and do not express CD4 or CD8 [202]. In pneumonia, the primary role of γδ T cells is early response to pathogens. Multiple signals stimulate these cells, including direct antigen binding to TCR, binding of PAMPs and DAMPs to TLRs or direct cytokine stimulation [203]. Activated γδT cells produce IL-17A and IFNγ, which promotes effector cell recruitment (macrophage and neutrophil), granuloma formation, and Th17 immune responses. Multiple lung pathogens induce γδ T cell numbers through cellular expansion [204–207]. The specific role of γδ T cells in pneumonia may be pathogen-dependent, as depletion of these cells results in decreased IFNγ production and inability to clear pathogen in *K. pneumoniae* and *S. pneumoniae* infections. In contrast, γδ T cell depletion improved pathogen clearance and increased IFNγ in a cryptococcus model [205] and increased IL-17 production and protection against fatal infection in an influenza model [208].

##### Invariant natural killer T cell (iNKT) cell

Invariant NKT cells (iNKT, also known as classical or type I NKT cells) express an αβ TCR with invariant α TCR and one of three β chains [209]. These cells respond to lipid antigens presented by the MHC class I-like molecule CD1d. These antigens include alpha-galactosylceramide (α-Gal-Cer) [210] and a variety of microbial- and host-derived lipids, allowing them to respond to a wide range of pathogens [211]. Of note, α-Gal-Cer loaded CD1d tetramers are a useful way to study human and mouse NKT cells [212]. NKT cells also respond directly to stimulation by the cytokines IFNβ, IL-1β, IL-12, IL-18, and IL-23 [213–215].

Sub-populations of NKT cells (NKT1, NKT2, and NKT17) are defined by their developmental programs, and cytokine repertoire expressed. Lungs are particularly enriched for NKT17 cells in the lung parenchyma, with lesser numbers of NKT1 and NKT2 cells in the marginated vascular pool [216]. NKT1 cells produce IFNγ and express the transcription factor T-box 21 (T-bet) [217, 218]. NKT2 cells produce interleukin-4 (IL-4), express PLZF (promyelocytic leukemia zinc finger protein) [218] and depend on PLZF, GATA binding protein 3 (GATA3), and interferon regulatory factor 4 (IRF4) for development [218, 219]. Finally, NKT17 cells produce IL-17 and express RORγt (retinoic acid receptor-related orphan receptor γ) [218, 220].

NKT cells play a critical role in the defense against multiple lung pathogens. They are required for clearance of respiratory bacteria such as *S. pneumoniae, P. aeruginosa, M. tuberculosis*, and *Chlamydia pneumoniae* [221–223], through recruitment of effector cells such as neutrophils and AMs via chemokine and cytokine production [221, 222]. NKT cells are also protective against viral pathogens such as influenza. Influenza A-infected DCs stimulate NKT cells, resulting in rapid production of interleukin-22 (IL-22) and modifying the activities of myeloid-derived suppressor cells and pro-inflammatory monocytes [224–226].

##### Mucosal-associated invariant T (MAIT) cells

Similar to NKT cells, Mucosal-associated invariant T cells (MAIT cells) also have a restricted TCR [227–229], but respond to the MR1 (MHC-class I-related) receptor, rather than CD1d. MR1 presents riboflavin (vitamin B_2_) metabolites that are produced by diverse bacterial and yeast species [228, 230–235]. In addition, MAIT cells can be stimulated by TCR-independent pathways, including IL-18 signaling [236, 237]. MAIT cells can also be subdivided by the cytokines that they produce. Thus far, MAIT1 (IFNγ-producing) and MAIT17 (IL-17 and IL-22 producing), but not MAIT2 have been identified. MAIT cells display heterogeneity in response to microbes and may function to distinguish between pathogen and commensal [227, 238]. Furthermore, MAIT cell numbers are reduced in germ-free mice, which is in contrast to iNKT cells, which are increased in these mice [239]. In the absence of infection, MAIT cell numbers in lungs of mice are low. However, cell numbers dramatically expand with infection by intracellular bacteria *Salmonella typhimurium*, *Francisella tularensis* and *L. pneumophila* [240, 241]. MAIT cells may play a protective role in infection, as their number is negatively correlated with disease severity and *P. aeruginosa* infection in cystic fibrosis [242], and MR1^−/−^ mice are more susceptible to *E. coli* and *Mycobacterium abscessus* [234]. Finally, humans recovered from severe influenza had higher numbers of MAIT cells in peripheral blood than those that died [237].

#### Innate lymphoid cells (ILCs)

Innate lymphoid cells (ILCs) are defined by a lymphoid morphology, but a lack of RAG-dependent antigen receptor, or cell-surface markers associated with other lymphoid and myeloid lineages [243]. Similar to the other innate-like lymphocytes discussed, parabiosis studies have demonstrated that ILCs are largely tissue resident and do not recirculate [244]. Instead of responding to antigen, they respond to direct stimulus by danger and stress signals. The first of these types of cells to be identified were natural killer (NK) cells and lymphoid tissue inducer (LTi) cells. Additional types of innate lymphoid cells have since been identified and ILCs are now categorized by the constellation of cytokines they produce. ILC1s produce type I cytokines (IFNγ and TNF), ILC2s produce type II cytokines (IL-4, IL-5, IL-9, and IL-13), and ILC3s produce IL-17 and IL-22. In the lung, the overwhelming proportion of ILCs are NK cells [245] and ILC2s [246], and are further discussed below.

##### Natural killer cells (NK)

NK cells are identified by the surface markers CD127^−^NKp46^+^Eomes^+^ and require the transcription factor T-bet [246]. NK cells express a wide variety of activating and inhibitory surface receptors that enable tuning of NK responses based on the combination of binding ligands [247, 248]. In the presence of activation signals, NK cells can directly kill target cells through the release of perforin and granzyme, recruit additional effector cells by the secretion of cytokines and chemokines or induce target cell apoptosis through the expression of apoptosis-inducing ligands such as Fas ligand (FasL) or TRAIL (TNF-related apoptosis-inducing ligand) [248, 249]. Conversely, NK cells can also produce anti-inflammatory cytokines. In the lung, where over-exuberant action of NK cells could be particularly deleterious, NK cells have a higher proportion of inhibitory to activating receptors, and tightly regulated activation [250]. However, NK cells are important during infection with *K. pneumoniae*, where they limit bacterial dissemination and improve survival, partially due to their production of IL-22 [251].

##### ILC2

ILC2 are defined by the markers CD90^+^CD127^+^RORγt^−^GATA3^hi^ [246]. They have been characterized as a “helper-type” ILC, and function in defense against helminth infection, as well as maintaining barrier integrity in the lung [248]. They are activated in response to IL-1β, prostaglandin D2, interleukin-22 (IL-22), interleukin-25 (IL-25), and thymic stromal lymphopoietin (TSLP), and once activated, they upregulate GATA3 to produce interleukin-5 (IL-5) and interleukin-13 (IL-13) [246]. In the lungs, their ability to produce large amounts of type II cytokines give them an important role in responding to viral and helminth infections, as well as in the pathogenesis of allergic asthma [252].

## Stromal cell defense

It has become clear that cells of the lung parenchyma are also active participants in immune responses. These cells also act as sentinels, producing cytokines and chemokines in response to PAMPs, and releasing DAMPs when injured, leading to the recruitment and activation of effector cell responses. During infection, parenchymal cells produce antimicrobial proteins that directly kill pathogens, and can modulate responses of resident and recruited leukocytes through chemokine, cytokine, and surface receptor expression. Finally, the unique structure of the lung and its parenchymal cells underlie lung-specific immune responses.

### Epithelial cells

Beyond providing a tight, physical barrier, lung epithelial cells in both the upper airway and alveoli play an active role in the initial defense against pathogens. The airways consist of a heterogenous population of epithelial cells with unique characteristics, making up a pseudostratified epithelial layer. The distal lung consists of two major epithelial cell types, the type I and type II alveolar epithelial cells (ATI and ATII, respectively). The thin, single-layer of ATI cells are primarily responsible for gas exchange, while ATII cells provide a regenerative reservoir for ATI cells and secrete pulmonary surfactant. Advances in our understanding of immune modulation by lung epithelial cells in the era of single-cell transcriptomics has recently been reviewed [253].

#### Airway epithelial cells

The upper airway epithelium is predominately made up of multiciliated, secretory and basal cells, with rarer subpopulations, such as ionocytes, neuroendocrine, tuft and goblet (rare in mouse, common in human) cells interspersed throughout [254]. The cooperate actions of ciliated and secretory cells provide the basis of the mucociliary escalator described in the previous section. Due to their location and important antimicrobial function, ciliated cells are often the primary targets of invading microorganisms, directly or indirectly interfering with mucociliary clearance. Cilia have more recently been shown to have chemosensing and signal transduction abilities to enhance innate immune defenses against pathogens, independent of direct clearance [255]. In 2009, it was discovered that bitter taste receptors (T2Rs) are expressed on cilia and when activated, increase ciliary beat frequency in a calcium-dependent manner [256]. Interestingly, T2R ligands, acyl-homoserine lactone and quinolones, are secreted by *P. aeruginosa* as quorum sensing molecules, resulting in increased nitric oxide production in air liquid interface cultures [257]. Taken together, these results suggest that ciliated cells may sense these molecules and enhance immune activity and ciliary beating in response to infection. In contrast, noncanonical activation of hedgehog signaling reduces cAMP levels in ciliated cells, resulting in lower ciliary beat frequency [258]. Multi-ciliated cells of the upper airway can also be distinguished due to their expression of a Piwi-like protein, MIWI2, the lack of which results in improved bacterial clearance during pneumococcal pneumonia due to increased expression of inflammatory proteins and recruitment of immune cells [259].

#### Alveolar epithelial cells

The distal alveoli are made up of type I and type II alveolar epithelial cells (ATI and ATII). ATI are flattened, squamous epithelial cells that are the primary location of gas exchange in the lung. ATII cells are cuboidal epithelial cells that produce pulmonary surfactant and other antimicrobial proteins, described previously. Importantly, ATII pneumocytes also provide a reservoir of progenitor cells, as they have the capacity to differentiate into type I cells if they are injured. This process is regulated by Wnt signaling in Axin^+^ type II cells, preventing their differentiation under homeostatic conditions and enhancing ATII proliferation following influenza-induced lung injury [260, 261]. Meanwhile BMP (bone morphogenic protein) signaling in ATII cells promotes type I differentiation, with underlying PDGFR-α (platelet-derived growth factor receptor-α) fibroblasts being the main source of both Wnt and BMP ligands [262].

While there is considerable evidence for the capacity of ATII cells to respond to lung infection, as evidenced by reduced bacterial killing in mice expressing a dominant negative form of IκB-α in type II cells [263], much less is known about the responsiveness of ATI cells. However, in response to pneumococcal pneumonia, ATI cells have been shown to produce CXCL5 (C-X-C motif chemokine ligand 5) in a RelA-independent manner [264]. Recently, expression of EMP2 (epithelial membrane protein 2) in ATI cells was shown to promote neutrophil influx following infection with *K. pneumoniae*. In this case, increased neutrophil levels worsened pneumonia outcome, and was dependent on the cholesterol binding properties of EMP2, which facilitated lipid raft formation, a known factor that improves PRR signaling [265]. These studies implicate the importance of ATI cells in the early response to infection, though many questions remain unanswered. Their fragility makes them especially difficult to work with, necessitating improved techniques for cellular isolation or in vivo analyses to thoroughly assess the role of ATI cells during pneumonia.

##### Direct killing

Airway epithelial cells produce the potent antimicrobial lysozyme, which directly hydrolyzes cell wall components of bacteria and lipocalin-2, a siderophore that limits the growth of *K. pneumoniae* and *E. coli* [266, 267]. Illustrating the importance of iron regulation during pneumonia, airway-epithelial derived hepcidin was recently shown to limit iron levels in the lungs by targeting the iron exporter, ferroportin. Hepcidin deficient mice had more severe pneumonia due to bacterial outgrowth and deficiency in macrophage function [268]. LL-37, the active C-terminal subunit of human cathelicidin, is also produced by airway and submucosal gland epithelial cells, directly contributing to bacterial cell death through membrane interactions. LL-37 also promotes both inflammatory activation by acting as a chemoattractant and immune modulation through sequestration of negatively charged molecules such as LPS and various DAMPs [269]. Epithelial cells can also synthesize components of the complement pathway that aid in defense against bacterial, viral and fungal pathogens [270]. SARS-CoV2 infected respiratory epithelial cells express multiple complement proteins in a JAK 1/2 (Janus kinase 1/2) dependent manner, including a functional C3 convertase [271]. While complement is beneficial in response to a multitude of lung infections, excessive complement activation has been associated with increased severity of SARS-CoV2 infection.

##### Roles in leukocyte recruitment

In addition to direct microbial inhibition, epithelial cells also promote leukocyte recruitment mainly via the direct or indirect production of chemokines and cytokines. Following pneumococcal pneumonia, conducting airway epithelial cells produce SECTM1 (secreted and transmembrane protein 1), which selectively binds to neutrophils in the infected lung, inducing them to produce CXCL2 and provide a feed-forward loop of neutrophil recruitment [272]. Moreover, with repeated exposure to pneumococcus, epithelial cells are reprogrammed to produce elevated levels of CXCL5 in a RelA and T-cell-dependent manner, which results in greater neutrophil recruitment at early time points post infection and more successful clearance of bacteria in comparison to naïve animals [273]. RelA signaling in epithelial cells also promotes CCL20 (C–C motif ligand 20) and GM-CSF expression [274]; the latter of which is largely protective against severe pneumonia [275], likely due to its myriad effects on neighboring AMs. GM-CSF is also protective against infection with *M. tuberculosis*, due to decreased levels of type I interferon-dependent neutrophil NET formation [276]. Moreover, *L. pneumophila* infected macrophages produce IL-1 which stimulates GM-CSF production in alveolar epithelial cells, leading to activation of recruited monocytes via increased glycolysis and cytokine production, the coordinate actions of which lead to improved control of infection [277]. Epithelial cells also express IL-17R and are highly responsive to multiple IL-17 family members. Following infection with *K. pneumoniae*, ablation of IL-17RA (interleukin-17 receptor A) in SCGB1A1 (secretoglobin family 1A member 1) expressing club cells significantly reduces CXCL5 levels, leading to reduced neutrophil recruitment and increased bacterial burden [278]. The IL-6 family cytokine, oncostatin-M (OSM), also enhances the expression of CXCL5 and neutrophil influx into the lungs after infection with *E. coli,* likely as a result of OSM-specific STAT3 (signal transduction and activator of transcription 3) activation in lung epithelial cells [279].

##### Repair and recovery

Epithelial cells also have substantive regenerative properties which are essential to recovery post-infection. While these regenerative properties are not aspects of innate immunity per-se, innate immune signals and programs can affect regeneration. For example, following influenza infection, epithelial-derived IL-33 promotes amphiregulin production in ILCs in the lung, facilitating repair [280]. In contrast, type I (IFN-α and IFN-β) and type III (IFN-λ) interferons induced following infection with influenza, interfere with epithelial repair and differentiation. Interferon signaling on epithelial cells leads to activation of p53, a prominent mediator of cell cycle arrest and apoptosis, which led to worsened outcomes to secondary infection with *S. pneumoniae* [281]. Similar results were seen during *K. pneumoniae* infection, where epithelial integrity was dampened by IFN-λ and protected by IL-22 expression [282]. The transcriptional coactivators, YAP (yes-associated protein 1) and TAZ (transcriptional coactivator with PDZ-binding motif) are negative regulators of the Hippo pathway, an evolutionary conserved pathway that regulates tissue development and regeneration [283, 284]. Recently, the activation of YAP/TAZ was investigated in the context of alveolar regeneration following pneumococcal infection, which primarily affects the lung alveoli. Here, the authors show that pneumonia-induced YAP/TAZ nuclear translocation promotes ATII to ATI differentiation between 7–14 days post-infection, the absence of which led to fibrotic lesions in the lung and slower recovery. Lung fibrosis was largely attributable to prolonged NF-κB signaling in mice lacking YAP/TAZ in ATII cells, as IκBα (NFκB inhibitor α) was discovered to be a direct target of YAP/TAZ and their associated transcription factor, TEAD (TEA domain family) [285]. Furthermore, ablation of ABL1 (Abelson kinase-1) in SCGB1A1-expressing secretory cells promotes the expansion of a small SCGB1A1/SPC (surfactant protein C) double positive population to replenish distal ATI cells that are injured during bacterial pneumonia. This rapid and enhanced proliferation limited alveolar edema and improved recovery following infection with *S. aureus* and *S. pneumoniae*, with no impact on bacterial burden or inflammatory cytokine response [286]. Given these results, greater emphasis should be placed on epithelial repair pathways as a protective mechanism against severe pneumonia as these pathways are largely applicable across diverse etiologies.

### Endothelial cells

At the alveolar-capillary interface, gas exchange is performed not only by the ATI cells as described above, but also lung microvascular endothelial cells, which provide red blood cells ready to transport oxygen. These cells also have several important purposes in response to respiratory infection. First and foremost, they are a primary conduit for leukocyte influx from the bloodstream, providing the tethering molecules involved in leukocyte transmigration. In response to inflammatory mediators, endothelial cells also release the neutrophil chemoattractant IL-8 in pre-formed granules, called Weibel-Palade bodies [287]. While the role of endothelial cells in leukocyte transmigration has been reviewed extensively elsewhere [288], including lung-specific mechanisms [181], much less is known about other direct and indirect endothelial responses to infection. At rest, endothelial cells provide a non-thrombogenic barrier to prevent inappropriate coagulation, however in response to inflammatory ligands, they activate platelets and produce coagulation components. Activation of endothelial cells during pneumonia also results in vasoconstriction and increased permeability leading to excessive alveolar edema [289]. Infection, including those caused by SARS-CoV2, elicit sizeable concentrations of pro-inflammatory cytokines, potentially resulting in life-threatening complications, such as cytokine release syndrome (CRS). Recently it was shown that endothelial trans-IL-6 signaling was required for maximum expression of IL-6, IL-8, CCL2 (C–C motif ligand 2) and PAI-1 (plasminogen activator inhibitor-1), all of which are increased in patients with CRS as a result of sepsis, ARDS, burns and SARS-CoV2 infection [290]. In contrast, sphingosine-1-phosphate receptor 1 (S1P1) receptor expression on endothelial cells may serve to limit overexuberant cytokine release, the agonism of which protected mice from lethal influenza infection through a reduction in endothelial-specific cytokine expression and leukocyte recruitment, with no impact on viral burden [291]. Endothelial injury that occurs during lung infection results in the recruitment and activation of platelets through endothelial-platelet interactions. Constraining platelet aggregation and activation was shown to prevent lung hemorrhage and thrombosis in mice infected with influenza in a manner that was dependent on a reduction in leukocyte recruitment and cytokine levels at 6 days post-infection. In this context, platelet activation was dependent on PAR4 (protease-activated receptor 4), which plays a role in thrombin-mediated platelet activation [292]. Direct activation of platelets has also been shown to occur during influenza infection through their phagocytosis of virions in a TLR7-dependent manner. Viral uptake stimulated platelets to produce complement component C3, which promoted neutrophil NETosis and myeloperoxidase production that was then tempered by GM-CSF production by the same activated platelets, likely as a mechanism for limiting inflammation and thrombosis [293].

It is important to note that lung endothelial cells are not a homogenous population, and their heterogeneity has recently been explored using single cell RNA-sequencing [294]. Five clusters of endothelial cells were identified, representing macrovascular endothelial cells, three clusters of microvascular endothelial cells, and a new Car4-high endothelial subset that lay juxtaposed to ATI cells following acute lung injury (influenza or bleomycin-induced). Although not specific to Car4-high endothelial cells, endothelial-epithelial interactions were predicted to occur between type I cells and multiple subpopulations of highly proliferative endothelial cells following influenza infection [294]. Just like epithelial cells, endothelial repair mechanisms are being recognized as an equally important process for limiting pneumonia-induced lung injury. COUP-TF2 (chicken ovalbumin upstream promoter–transcription factor 2) has been identified as a transcription factor that is critical to endothelial cell proliferation through its activation of the cell cycle protein cyclin D1 (CCND1), and promotion of angiogenesis through VEGFA/VEGFR2 (vascular endothelial growth factor A/vascular endothelial growth factor receptor 2)-mediated activation of neuropilin-1 [295]. Deletion of COUP-TF2 reduced the proliferative capacity of endothelial cells ex vivo and in vivo, resulting in exacerbated lung injury and increased mortality in influenza infected mice [295]. Interestingly, this study corroborates the previous finding that surviving endothelial cells located near areas of epithelial injury exhibit the greatest capacity for proliferation. These studies highlight the significance of structural cells, such as endothelial, epithelial cells and fibroblasts, in contributing to host defense and protection from pneumonia-induced lung injury. Current studies in endothelial cells are largely limited to that which occurs following viral infection (influenza, SARS-CoV2) and future studies should expand to infection with bacterial or fungal pathogens which may demonstrate the broad relevance of endothelial-specific mechanisms during lung infection.

### Fibroblasts and the extracellular matrix

The primary function of the pulmonary extracellular matrix (ECM) is to anchor and provide structural support for the lung epithelium and endothelium. The components of the ECM are synthesized and maintained in large part by fibroblasts, with minor contributions from endothelial and epithelial cells. Broadly, the ECM can be subdivided into the interstitial matrix and basement membranes [296], together comprising 150 core structural proteins in the lung [297, 298]. The basement membrane is comprised of glycoproteins in a thin layer on the basal side of cells, while the interstitial matrix is a loose meshwork of fibrillar collagens (I, II, III, V, XI), elastic fibers (crosslinked elastin with outer layer of microfibrils), fibrillins, glycoproteins, proteoglycans (PGs) and glycosaminoglycans (GAGs) located between the basement membranes.

In the lung, the GAG hyaluronan (HA) and HA-bound proteoglycans (PGs) are structural members of the interstitial matrix and important regulators of immune responses in the lungs. In the lung at baseline, HA is mostly synthesized as high molecular weight HA (HMW HA), which functions to maintain lung homeostasis and limit inflammatory responses [299]. PGs are comprised of a central protein core (in the lung most often versican, biglycan and decorin), crosslinked to negatively charged GAG side chains (such as chondroitin sulfate or heparin sulfate) or HMW HA. This complex matrix structure along with the negative charge of GAG chains forms a hydrated gel surrounding the other structural components of the ECM (collagen and elastin), as well as multiple soluble proteins, including chemokines, cytokines, growth factors, and matrix-modifying proteins [300]. The complexity of this structure allows sequestration of these various proteins, allowing for rapid, yet tight control of immune responses.

During injury or inflammation HA is cleaved into low molecular weight HA (LMW HA) through direct action of hyaluronidases, ROS or tissue injury. LMW HA are highly pro-inflammatory and can induce the production of pro-inflammatory cytokines and chemokines leading to increased cell recruitment [299]. In addition, neutrophil chemokines bind lung GAGs with varying affinity, generating functionally different chemokine gradients to coordinate neutrophil migration speeds through the ECM [301]. The production of the PG versican increases in acute and chronic inflammation, leading to sequestration and stabilization of cytokines and chemokines, activation of TLR signaling and modification of overall ECM structure to affect cell migration [85]. In an influenza model of pneumonia, damage-responsive fibroblasts produced the protease ADAMTS4 (a disintegrin and metalloproteinase with thrombospondin motifs 4), leading to the cleavage of versican, enabling immune cell infiltration [302]. Overall, the ECM plays an integral role in innate immune responses, yet our understanding of the complex interplay of ECM components and the contribution of the surrounding cells is in its infancy.

## Conclusion

The innate immune response in the lung is a complex and sophisticated rapid-response arm of the immune system. Given the rapid and early nature of innate immune responses, it is likely that the future of pneumonia therapeutic discovery will involve well-calibrated and personalized modulation of these responses to maximize pathogen clearance while minimizing secondary tissue injury. While many aspects of the innate immune system have been broadly established for some time, two recent advances in the field have radically changed our understanding of this system in the lung. The advent of single-cell based technologies has revealed variations and heterogeneity in innate immune responses that are organ- and cell-type- specific. These new single-cell “omics” databases provide huge amounts of lung-specific data related to innate immunity. The second major advancement relates to the recent SARS-CoV-2 pandemic, where the collection of clinical samples and data led to an unprecedented level of granularity regarding both innate and adaptive immune responses to a single pulmonary pathogen. As yet, the full impact of these new sources of information on our understanding of innate immunity has not yet been felt. It is our hope that these two bodies of data will provide the basis for countless insights into the biology of lung innate immune responses, and lead to novel immune modulatory therapeutics to treat pneumonia.

## Data Availability

Not applicable.

## Abbreviations

*A. baumannii*: *Acinetobacter baumannii*
ABL1: Abelson kinase-1
ADAMTS4: A disintegrin and metalloproteinase with thrombospondin motifs 4
AIM2: Absent in melanoma 2
α-Gal-Cer: Alpha-galactosylceramide
ALR: AIM2-like receptors
AM: Alveolar macrophage
ARDS: Acute respiratory distress syndrome
ASC: Apoptosis-associated speck-like protein containing a CARD
ATI: Alveolar epithelial type I
ATII: Alveolar epithelial type II
BMP: Bone morphogenic protein
CARD: Caspase recruitment domain
CCL20: C-C motif ligand 2
CCL20: C-C motif ligand 20
CCND1: Cyclin D1
CCR2: C-C motif chemokine receptor 2
CD5L: CD5 molecule-like
cGAS: Cyclic GMP-AMP synthase
COUP-TF2: Chicken ovalbumin upstream promoter-transcription factor 2
CR: Complement receptor
CRS: Cytokine release syndrome
CXCL1: C-X-C motif chemokine ligand 1
CXCL2: C-X-C motif chemokine ligand 2
CXCL5: C-X-C motif chemokine ligand 5
DAMP: Damage-associated molecular pattern
*E. coli*: *Escherichia coli*
ECM: Extracellular matrix
EMP2: Epithelial membrane protein 2
*F. tularensis*: *Francisella tularensis*
FABP4: Fatty acid binding protein 4
FasL: Fas ligand
FcR: Fc receptor
GAG: Glycosaminoglycan
GM-CSF: Granulocyte-macrophage colony-stimulating factor
*H. influenzae*: *Haemophilus influenzae*
HA: Hyaluronan
HIF-1α: Hypoxia inducible factor 1 alpha
IαI: Inter-alpha-inhibitor
IFI16: Interferon gamma inducible protein 16
IFI204: Interferon gamma inducible protein 204
IFN-β: Interferon-beta
IFN-γ: Interferon gamma
IFNGR1: Interferon gamma receptor 1
IgA: Immunoglobulin A
IgG: Immunoglobulin G
IκBa: NFκB inhibitor alpha
IL-1: Interleukin-1
IL-1beta: Interleukin-1 beta
IL1-Ra: Interleukin-1 receptor antagonist
IL-4: Interleukin-4
IL-5: Interleukin-5
IL-6: Interleukin-6
IL-8: Interleukin-8
IL-10: Interleukin-10
IL-13: Interleukin-13
IL-17A: Interleukin-17A
IL-17RA: Interleukin-17 receptor A
IL-18: Interkeukin-18
IL-22: Interleukin-22
IL-23: Interleukin-23
IL-25: Interleukin-25
ILC: Innate lymphoid cell
IM: Interstitial macrophage
Inkt: Invariant natural killer T cell
IRF3: Interferon regulatory factor 3
IRF4: Interferon regulatory factor 4
JAK1/2: Janus kinase 1/2
*K. pneumoniae*: *Klebsiella pneumoniae*
*L. pneumophila*: *Legionella pneumophila*
LGP2: Laboratory of genetics and physiology 2
LIF: Leukemia inhibitory factor
LPS: Lipopolysaccharide
LTA: Lipoteichoic acid
LTi: Lymphoid tissue inducer
*M. abscessus*: *Mycobacterium abscessus*
*M. tuberculosis*: *Mycobacterium tuberculosis*
MAIT: Mucosal-associated invariant T cells
MARCO: Macrophage receptor with collagenous structure
MAVS: Mitochondrial antiviral signaling protein
MCL-1: Myeloid cell leukemia 1
mCRAMP: Cathelicidin-related antimicrobial peptide
MDA5: Melanoma differentiation-associated protein 5
MHCII: Major histocompatibility complex II
MR1: MHC-class I-related
MRSA: Methicillin resistant *S. aureus*
MUC1: Mucin 1
MUC5AC: Mucin 5AC
MUC5B: Mucin 5B
NET: Neutrophil extracellular trap
NFκB: Nuclear factor kappa B
NK: Natural killer
NLR: Nod-like receptor
NOD: Nucleotide-binding oligomerization domain
OSM: Oncostatin M
*P. aeruginosa*: *Pseudomonas aeruginosa*
PAI-1: Plasminogen activator inhibitor-1
PAMP: Pathogen-associated molecular pattern
PAR4: Protease-activated receptor 4
PDGFR-α: Platelet-derived growth factor receptor alpha
PG: Proteoglycan
PLZF: Promyelocytic leukemia zinc finger protein
PPARγ: Peroxisome proliferator-activated receptor gamma
PRR: Pattern recognition receptor
RIG-I: Retinoic acid-inducible gene I
RLR: RIG-I-like receptors
RORγT: Retinoic acid receptor-related orphan receptor gamma
RSV: Respiratory synctial virus
s-IgA: Secretory IgA
*S. aureus*: *Staphylococcus aureus*
*S. pneumoniae*: *Streptococcus pneumoniae*
*S. typhimurium*: *Salmonella typhimurium*
SARS-CoV: Severe acute respiratory coronavirus
SCGB1A1: Secretoglobin family 1A member 1
SECTM1: Secreted and transmembrane protein 1
SIRP-α: Signal regulatory protein alpha
SOCS3: Suppressor of cytokine signaling 3
SP-A: Surfactant protein A
SP-C: Surfactant protein C
SP-D: Surfactant protein D
SR-A: Scavenger receptor-A
SR-BI: Scavenger receptor class B type I
STAT2: Signal transducer and activator of transcription 2
STAT3: Signal transducer and activator of transcription 3
STING: Stimulator of interferon genes
T-bet: T-box 21
T2R: Bitter taste receptor
TAZ: Transcriptional coactivator with PDZ-binding motif
TCR: T cell receptor
TEAD: TEA domain family
TGF-β1: Transforming growth factor-beta1
TIR8: Toll/interleukin-1 receptor 8
TLR: Toll like receptor
TNF-α: Tumor necrosis factor alpha
TNFAIP6: TNF-alpha induced protein 6
TRAIL: TNF-related apoptosis-inducing ligand
TSLP: Thymic stromal lymphopoietin
VEGFA: Vascular endothelial growth factor A
VEFR2: Vascular endothelial growth factor receptor 2
YAP: Yes-associated protein 1
